# Think Unique: Perceptions of Uniqueness Increases Resistance to Persuasion and Attitude-Intention Relations

**DOI:** 10.3389/fpsyg.2021.653031

**Published:** 2021-05-14

**Authors:** Kevin L. Blankenship, Kelly A. Kane, Marielle G. Machacek

**Affiliations:** Communication Studies Program, Department of Psychology, Iowa State University, Ames, IA, United States

**Keywords:** uniqueness, persuasion, attitude strength, intentions, attitudes, resistance

## Abstract

The present research examines whether the perceived uniqueness of one’s thoughts and salience of uniqueness motivations can influence attitude strength and resistance. Participants who rated their thoughts as relatively unique formed attitudes that showed greater correspondence with behavioral intentions to act on the attitude (Study 1). In Study 2, participants who recalled a previous purchase motivated by the desire to be unique (versus to fit in) after generating message counterarguments were less persuaded (more resistant) and reported greater willingness to act on their (negative) attitude. Moreover, attitudes mediated the effect of the purchase manipulation on intentions to act on the attitude.

## Introduction

When exposed to a persuasive message, individuals bring many aspects of themselves into the context, such as pre-message attitudes (Sherif and Sherif, 1967), topic relevance (Apsler and Sears, 1968), and a myriad of individual difference variables (Briñol and Petty, 2005). Thus, given the amount and complexity of variables that affect persuasion, it is no surprise that (lack of) attitude change or persuasion is multiply determined (Chaiken and Trope, 1999). The current research focuses on the role of uniqueness—the experience of being original or different from others—in persuasion. Specifically, we present two studies that examine how perceived uniqueness can affect resistance to persuasion and behavioral intentions, two outcomes associated with an attitude’s durability and impact (i.e., strength; Krosnick and Petty, 1995).

## Uniqueness and Persuasion

Uniqueness theory (Snyder and Fromkin, 1980) posits that, while individuals strive to fit in, they also seek to maintain a sense of self as distinct (Brewer, 1991) that drives individuals to be different from others (Maslach, 1974). Uniqueness is viewed as a positive form of nonconformity (Lynn and Harris, 1997), particularly in Western cultures (Markus and Kitayama, 1991). Indeed, individuals high in self-reported uniqueness tend to be more resistant to persuasion (Lynn and Harris, 1997), particularly when the message position is supported by a numerical majority (Imhoff and Erb, 2009), or if resistance helps maintain a sense of uniqueness (Snyder and Fromkin, 1980). Moreover, a common measure of individuals’ differences in uniqueness, the Need for Uniqueness scale (Snyder and Fromkin, 1977) contains items that make explicit the link between uniqueness and nonconformity or resistance. For example, the scale includes items such as “*As a rule, I strongly defend my own opinions.*” Similarly, the Consumers’ Need for Uniqueness Scale (Tian et al., 2001) contains a counterconformity factor that includes items such as “*I enjoy challenging the prevailing taste of people I know by buying something they wouldn’t seem to accept.”* Thus, to the extent that these measures demonstrate content validity, there is an inherent connection between uniqueness and resistance to influence (Tepper and Hoyle, 1996).

Although resistance to persuasion may be an aspect of uniqueness, the Need for Uniqueness scale could also be measuring resistance in addition to a general motivation for uniqueness, which may not be a necessary component of uniqueness effects on persuasion (Hmel and Pincus, 2002). Viewing oneself as different from others seems conceptually distinct from being generally motivated to defend one’s opinions. What is more, the utility of uniqueness on attitudes and intentions may make salient goals that vary in their cultural relevance (Markus and Kitayama, 1991). That is, Western cultures embrace standing out as an individual more so than Eastern cultures do, likely because these cultures value and reward individualistic thinking and behavior. However, both individualistic and collectivistic goals can differ in salience within a particular culture (Gardner et al., 1999). Is the perception of uniqueness exhibited in one’s thoughts or motivations able to create a strong (impactful) or resistant (durable) attitude? These studies will examine this question in detail.

In addition to viewing oneself as unique, an individual can view thoughts and motivations as unique; these views are likely to be consequential in persuasion. Contemporary dual- and multi-process models of persuasion posit that one’s thoughts about an attitude object play an important role in the durability of judgments and attitudes, particularly when one is motivated and able to process a message (Chaiken and Trope, 1999). Moreover, it is under these conditions that thoughts have the greatest potential to affect attitudes and judgments.

Decades of persuasion research has found that not all thoughts generated in a persuasive context have equal weight in forming judgments; they vary in terms of their favorability (Petty et al., 1981) and confidence (Wagner et al., 2012). One dimension relevant to the current research is that of perceived uniqueness, or the perceived originality of a thought. Because one’s thoughts can be viewed as a personal possession (Abelson, 1986), perceiving one’s thoughts as unique may be associated with less persuasion following exposure to a counterattitudinal message. Indeed, unique arguments tend to be more persuasive than familiar arguments (Vinokur and Burnstein, 1978). Therefore, the perceived uniqueness or novelty of a thought may be an important dimension to consider in persuasion.

Moreover, it may be that simply making salient one’s uniqueness as an individual may alter responses to persuasive messages. For instance, increasing the salience of one’s own uniqueness may reduce the persuasiveness of a counterattitudinal messageTherefore, making readers’ uniqueness motivations salient after reading and processing a counterattitudinal message may lead them to have less favorable attitudes (i.e., increased resistance to change) and increase intentions to act on those resistant attitudes. Indeed, making uniqueness salient leads to an increase in attitude certainty (a form of attitude strength), as well as increased attitude-intention correspondence when that motivation was validated (Clarkson et al., 2013). However, in addition to examining uniqueness motivations and not individual difference measures of uniqueness, the current studies also focus on perceptions of thought uniqueness.

## Research Overview

We present two studies that examine the role of uniqueness on attitude strength in a persuasion context. Specifically, we examine whether perceived thought uniqueness increases attitude-intention relations (a feature of an impactful attitude), and whether activating a uniqueness mindset can increase attitudes’ resistance to change (Krosnick and Petty, 1995).

Study 1 used a correlational design: participants rated their own thoughts about a counterattitudinal message for novelty and originality, then reported their attitudes and behavioral intentions toward the attitude issue. We hypothesize that perceived uniqueness of one’s thoughts will increase intentions to act against the message. In Study 2, we manipulate the salience of uniqueness motivations to test whether uniqueness salience affects resistance and intentions. We expect to demonstrate that making uniqueness salient after participants report their thoughts about a counterattitudinal message consequentially affects attitude strength, specifically it can induce less favorable attitudes in response to a counterattitudinal message (i.e., resistance).

## Study 1

### Methods

#### Participants and Procedure

The key prediction is that self-reported thought uniqueness would moderate the relation between attitudes and intentions. A power analysis was conducted based on the effect size observed in previous research examining moderators of attitude-intention correlations. Kraus (1995) reported an average effect size of attitude-intention moderators from 12 studies and found a medium effect. Therefore, we examined the statistical power of our study to detect an effect of this size (Cohen’s *q* = 0.39; medium effect, Cohen, 1988) using G^∗^Power (Faul et al., 2007), which indicated that the desired sample size for a two-tailed test (α = 0.05) with.80 power is *N* = 46. We chose to attain more power by collecting data until the end of the semester, which increased the final sample size.^1^ As a result, 91 undergraduate students from a large Midwestern university (39 male, 52 female; *M*_age_ = 19.35, *SD*_age_ = 1.69; 91% Caucasian) participated in exchange for course credit. Data from three participants were excluded because they did not complete the thought listing task, reducing the final sample to 88 participants.

Participants completed the study on psychology lab computers. After the informed consent process, participants read a proposal to implement comprehensive final exams at their university the following academic year; the cover story specified that the university wanted feedback from students about the proposal. Specifically, participants were instructed to write arguments against the proposed exam. Afterward, the computer presented participants’ own arguments to them, and participants were instructed to rate each argument in terms of how novel and original it was. Following the ratings, participants reported their attitudes toward the exam policy and willingness to act on those attitudes. Afterward, participants completed demographic measures and were debriefed as to the fictitious nature of the exam policy.^2^

#### Study Variables

**Counterarguments.** After reading the proposal, participants completed a guided elaboration task, such that they were instructed to list up to four counterarguments to the proposal. These types of instructions are effective at inducing resistance to persuasion (Killeya and Johnson, 1998). To assess whether the thoughts were indeed opposing the proposal, research assistants rated each thought as favoring, opposing, or indicating neutrality toward the proposal. We subtracted the number of opposing thoughts from the number of favorable thoughts and then divided the difference by total number of thoughts. Negative numbers suggest greater counterargumentation and therefore opposition to the proposal (Wegener et al., 1995).^3^ Research assistants also rated thoughts in terms of how convincing/strong they were on a 9-point scale (1 = *not at all convincing*, 9 = *extremely convincing*).

**Counterargument Uniqueness.** After completing the writing task, participants were presented their counterarguments and asked to rate each on two 7-point scales for originality (defined to participants as something only they would think of; 1 = *not at all*; 7 = *very*) and uniqueness (1 = *not at all*; 7 = *very*). Separate originality and uniqueness ratings were calculated for each participant by averaging the ratings across the counterarguments provided. Originality and uniqueness ratings were correlated (*r* = 0.57) and were combined to create a composite uniqueness index. Higher scores indicate greater perceived uniqueness.

**Attitudes Toward Issue.** After rating their counterarguments, participants reported their attitudes toward comprehensive final exams on six 9-point Likert-type scales (1 = *bad, disagree, foolish, harmful, unfavorable*, and *do not approve*, 9 = *good, agree, wise, beneficial, favorable*, and *very much approve*, respectively; α = 0.96). Higher scores indicate more favorable attitudes toward the proposal (and therefore less resistance).

**Intentions.** Participants then reported their intentions toward the proposal and willingness to act on the attitude on six 9-point scales. Participants reported their willingness to discuss their attitude toward the policy with someone who has an opposing viewpoint, discuss their attitude in public, and sign a petition that supports their attitude toward the policy (1 = *not at all*; 9 = *very*). Participants were then instructed to imagine that the university allowed students to vote on the proposal and to respond to three intention statements related to voting against the proposal, voting “no” on the proposal, and voting to support the proposal (reverse scored) on a 9-point scale (1 = *strongly disagree*, 9 = *strongly agree*). The six items showed acceptable reliability (α = 0.73) and were combined to create an index of intentions. Higher scores indicate greater intentions to act on one’s attitude.

#### Results

Table 1 presents the descriptive statistics for the variables of interest. Participants generated an average of 2.9 counterarguments (*SD* = 0.97), with the mean favorability *M* = −0.38, *SD* = 0.26), indicating that participants generated unfavorable thoughts, as expected. The average strength of the counterarguments, as judged by research assistants, was *M* = 3.15 (*SD* = 1.32) on a 9-point scale and was not correlated with counterargument uniqueness.

**TABLE 1:** Study 1 Descriptive statistics for the variables of interest.

	**Mean (SD)**	**2.**	**3.**	**4.**	**5.**	**6**	**.7**	**8.**
1. #CA	2.88 (0.97)	0.58*	–0.15	0.25*	–0.19	0.31*	–0.06	0.11
2. TFAV	−0.38 (0.25)	–	−0.35*	0.16	0.07	0.12	–0.2	0.02
3. CASTR	3.15 (1.32)		–	0.16	–0.1	0.17	0.09	0.15
4. UNIQ	4.1 (1.25)			–	–0.07	0.17	–0.09	–0.02
5. ATT	4.45 (1.93)				–	−0.6*	0.003	–0.1
6. INTEN	5.71 (1.64)					–	–0.004	0.14
7. AGE	19.36 (1.71)						–	0.37*
8. GEN	–							–

*p < 0.05. #CA, number of counterarguments; TFAV, thought favorability; CASTR, Counterargument strength; UNIQ, self-reported thought uniqueness index; ATT, attitudes; INTEN, intentions to act on one’s attitude; AGE, participant age; GEN, participant gender.

We hypothesized that those who judged their counterarguments as more novel would exhibit a stronger relation between reported attitudes and behavior intentions. Because the proposal is meant to be counterattitudinal, we would expect more negative attitudes to predict greater intentions to act on the attitude. Specifically, negative attitudes supported by highly unique counterarguments would lead to increased intentions to act than attitudes supported by less unique counterarguments.

To test this hypothesis, we conducted a centered regression using the PROCESS macro for SPSS (Model 1; Hayes, 2013), with participants’ intentions being predicted by participants’ counterargument uniqueness scores, attitudes toward the policy, and their interaction. Self-rated counterargument uniqueness was positively associated with willingness to act on one’s attitude, *b* = 0.23, *SE* = 0.11, *t*(84) = 2.04, *p* = 0.045, 95% *CI* [0.006, 0.45], *d* = 0.22. Participants’ attitude valence was negatively associated with intentions to act on the attitude: greater negativity was associated with greater intentions, *b* = −0.45, *SE* = 0.07, *t*(84) = −6.11, *p* < 0.001, 95% *CI* [−0.6, −0.3], *d* = 0.7. The predicted interaction was also significant, *b* = −0.12, *SE* = 0.05, *t*(84) = −2.47, *p* = 0.02, 95% *CI* [−0.22, −0.02], *f*^2^ = 0.14.

Specifically, at 1 *SD* below the mean of counterargument uniqueness, more negative attitudes were associated with greater intentions to act, *b* = −0.3, *SE* = 0.11, *t*(84) = −2.76, *p* = 0.007, 95% *CI* [−0.51, −0.08], *d* = 0.3. However, at 1 *SD* above the mean of counterargument uniqueness, attitudes predicted willingness to a greater degree, such that more negative attitudes were associated with increased willingness to act on the attitude, *b* = −0.6, *SE* = 0.08, *t*(84) = −7.3 *p* < 0.001, 95% *CI* [−0.77, −0.44], *d* = 0.82. As self-rated perceptions of counterargument uniqueness increased, attitudes were more strongly correlated with intentions.^4^

#### Discussion

Study 1 provided initial evidence of counterargument uniqueness as moderator of attitude-intention correspondence. Participants who rated their counterarguments as relatively unique reported intentions toward a counterattitudinal proposal that corresponded to their attitudes (a type of attitude strength; Krosnick and Petty, 1995).

Having provided correlational evidence in Study 1, we sought to extend the findings in two ways. First, Study 2 introduces a manipulation of uniqueness salience after participants report their counterarguments. After reporting their counterarguments, participants recalled a time where they purchased something in order to fit in or to be unique. Similar manipulations have been used to make salient various metacognitive experiences and motivations (Petty et al., 2007). Such a paradigm mirrors that of Study 1, where uniqueness salience was activated after counterargument generation. Whereas Study 1 examined the moderating effect of perceived thought uniqueness on attitudes as driving intentions, Study 2 manipulates the extent to which the uniqueness motivation was salient at all. Therefore, In Study 2, we changed the relative value of uniqueness to participants before they ever generated counterarguments. Through changing the paradigm to make uniqueness motivations salient before (rather than after) the generation of counterarguments, we could therefore examine the causal influence of uniqueness on counterarguments and attitudes.

Second, because we were interested in effortful counterargument generation, we added an index of elaboration by manipulating the convincingness of the message arguments (Petty and Cacioppo, 1986). Previous research has demonstrated different processes underpin effortful and non-effortful resistance strategies (Wegener et al., 2004). Study 2 examined resistance through having participants read one of two versions of the comprehensive exam message. One contained relatively weak support for the policy, and one provided relatively strong support. Previous research has demonstrated that individuals are better able to discern strong support for a position from weak support, but only when they are motivated and able to process the message (Petty and Wegener, 1998). Thus, the argument quality manipulation will help serve as a check to ensure that participants are indeed effortfully processing the message. In particular, lack of an effect of argument quality on attitudes would indicate that any differences in attitudes and intentions across the uniqueness manipulation were due to relatively low effort processes, whereas a main effect would indicate more effortful processes at play.

## Study 2

### Methods

#### Participants and Procedure

While we were predicting a main effect of uniqueness on attitudes and intentions, we chose to have enough power (at least 80%) to test for any interaction in the factorial design. The average persuasion variable x argument quality effect on attitudes is *r* = 0.4 (Carpenter, 2015), an effect size we used with G^∗^Power (Faul et al., 2007), which recommended *N* = 199 for a two-tailed test (*α* = 0.05) with 0.80 power. We again used a time-based stopping rule by collecting data until the end of the semester. Thus, 238 undergraduate students from a large Midwestern university (109 male, 123 female; *M*_age_ = 19.18, *SD*_age_ = 1.42; 86% Caucasian) participated in a 2(Purchase: common vs. unique) X 2(Argument quality: weak vs. strong) between-participants design. They were compensated with course credit. Data from four participants were excluded because they did not write anything during the thought-listing task, thus reducing the final sample to 234 participants.

Participants again completed all measures on psychology lab computers. After indicating their consent, participants received the same cover story as in Study 1. Participants then read one of two versions of the comprehensive exam message (with weak or strong arguments), and then wrote counterarguments. Afterward, participants were exposed to the uniqueness manipulation, and then participants reported their attitudes toward comprehensive exams and voting intentions associated with the proposal.

#### Independent Variables

**Argument Quality.** Participants read a message containing four arguments that provided either relatively weak support (e.g., “*taking the comprehensive exams is a fair practice*”) or provided relatively strong support (e.g., “*students have a greater chance of earning more money if the policy is implemented*”; see Petty and Cacioppo, 1986 for similar arguments) for the policy.

**Purchase Uniqueness.** After the counterargument-listing task, participants completed an ostensibly unrelated memory task where they reported an instance when they purchased a product. Participants in the unique condition were instructed to recall an instance when they purchased a product to be unique and “stand out.” Participants in the common condition were instructed to recall an instance when they purchased a product to “fit in.” Across both conditions, the most frequently mentioned products were apparel items (e.g., shoes, sweatshirts). Following the memory task, participants reported how descriptive they were, how clearly they were able to recall the purchase, and how much they enjoyed describing the memory (1 = *not at all*; 5 = *very much*). Descriptiveness and clarity were not influenced by either of the manipulations or the interaction (*p*s > 0.4). Participants did, however, report greater enjoyment recalling a unique purchase (*M* = 3.07, *SD* = 1.12) than a common purchase (*M* = 2.67, *SD* = 0.93), *F*(1, 230) = 8.57, *p* = 0.004, *d* = 0.38.^5^

#### Dependent Variables

Participants completed the same guided elaboration task, attitude measure (α = 0.96), and intentions to act (α = 0.74) using the same scales as in Study 1. Research assistants rated thoughts’ strength and persuasiveness on a 9-point scale (1 = *not at all convincing*, 9 = *extremely convincing*).

#### Results

Similar to Study 1, participants generated an average of 2.5 counterarguments (*SD* = 0.81), with the mean favorability *M* = −0.42 (*SD* = 0.26), suggesting that participants were generating unfavorable thoughts. The average strength of the counterarguments was *M* = 3.37 (*SD* = 1.05). Neither thought favorability nor the counterargument strength measure were affected by the purchase manipulation, argument quality, nor their interaction (*p*s > 0.12).^6^

**Attitudes.** A 2(Purchase: common vs. unique) X 2(Argument quality: weak vs. strong) between-participants factorial Analysis of Variance (ANOVA) revealed that participants had more favorable attitudes toward the proposal after reading strong (*M* = 4.81, *SD* = 1.79) than weak arguments (*M* = 3.8, *SD* = 1.77), *F*(1, 230) = 20.56, *p* < 0.001, *d* = 0.59, suggesting that participants were effortfully processing the counterattitudinal message. More germane to the hypotheses, recalling a unique purchase led to more negative attitudes toward the proposal (*M* = 4.04, *SD* = 1.76) than recalling a purchase to fit in did (*M* = 4.54, *SD* = 1.9), *F*(1, 230) = 5.84, *p* = 0.02, *d* = 0.35. The interaction was not significant, *F*(1, 230) = 0.74, *p* = 0.39, *d* = 0.11.

**Intentions.** A factorial ANOVA on the intentions measure revealed that participants reported lower intentions to act on their counterattitudinal opinions after reading strong (*M* = 5.65, *SD* = 1.57) than weak arguments (*M* = 6.24, *SD* = 1.46), *F*(1, 230) = 9.4, *p* = 0.002, d = 0.41. Importantly, recalling a unique purchase led to greater intentions to act (*M* = 6.16, *SD* = 1.54) than recalling a purchase to fit in (*M* = 5.73, *SD* = 1.52), *F*(1, 230) = 4.98, *p* = 0.03, d = 0.29. The Purchase x Argument quality interaction was not significant, *F*(1, 230) = 0.01, *p* = 0.91, d = 0.06.

**Uniqueness Moderation of Attitude-Willingness Link.** Because attitudes and intentions were reported after the uniqueness manipulation, we examined, as in Study 1, whether the correlation between attitudes and intentions was stronger when participants recalled a unique purchase rather than a purchase to fit in. We conducted a centered regression using the PROCESS macro for SPSS (Model 1; Hayes, 2013), with participants’ intentions being predicted by the uniqueness manipulation (coded as −1 = common; 1 = unique), attitudes toward the policy, and the interaction between uniqueness and attitudes. We found attitudes were associated with willingness to act on one’s attitude, *b* = −0.52 *SE* = 0.04, *t*(230) = −12.04, *p* < 0.001, 95% *CI* [−0.61, −0.43], *d* = 0.86. The interaction was not significant, *b* = −0.05, *SE* = 0.04, *t*(230) = −1.19, *p* = 0.24, 95% *CI* [−0.14, 0.03], *d*, = 0.08. However, the pattern was consistent with Study 1, such that attitudes were a better predictor of intentions in the purchase conditions *b* = −0.57, *SE* = 0.06, *t*(114) = −9.28, *p* < 0.001, 95% *CI* [−0.7, −0.45], *d* = 0.89, than in the fit in conditions *b* = −0.47, *SE* = 0.06, *t*(116) = −7.79, *p* < 0.001, 95% *CI* [−0.59, −0.35]. *d* = 0.73.^7^

**Mediation Analyses.** We expected that, consistent with previous research on attitude strength (e.g., Kraus, 1995), participants’ attitudes would mediate the effect of uniqueness on intentions. That is, recalling a unique purchase would lead to more negative attitudes, which would then increase intentions to act on the (negative) attitude. To test this, we conducted a mediational analysis using bootstrapping procedures using the PROCESS macro for SPSS (Model 4; Hayes, 2013). The uniqueness manipulation (coded as −1 = common; 1 = unique) was treated as the distal variable, and participants’ attitudes were treated as a potential mediator. The bootstrapping analyses randomly drew cases from the sample data (with replacement) and created 5000 bootstrap data sets of equal size to the original sample. Each data set supplied an estimate of the indirect (mediational) effect of the potential mediator. Using these estimates, confidence intervals were created to examine whether the population value of each indirect effect differed from zero.

**FIGURE 1:**
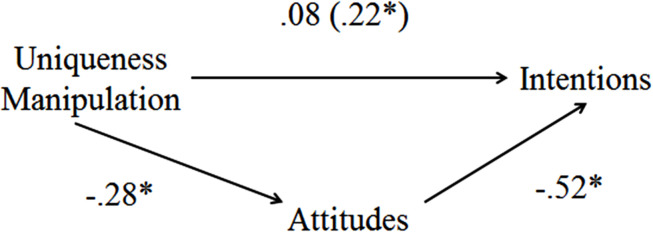
Study 2 mediation model showing the effect of uniqueness on intentions, as mediated through attitudes.

The indirect effect of participants’ attitudes (*M* = 0.13, *SE* = 0.06) mediated the effect of the uniqueness manipulation on intentions, 95% BS *CI* [0.01, 0.26] (see Figure 1). In addition, the uniqueness effect on intentions was no longer significant, *b* = 0.07, *SE* = 0.08, *t* = 0.93, *p* = 0.35, 95% *CI* [−0.08, 0.23]. Thus, participants who recalled a purchase meant to make them be unique had more negative attitudes toward the counterattitudinal message, which then led to greater intentions to act on their (negative) attitudes. Such a pattern is consistent with uniqueness salience increasing attitude strength by inducing lack of persuasion and guiding intentions to act on one’s attitude.^8^

## General Discussion

Using correlational (Study 1) and experimental (Study 2) designs, the current studies demonstrate that individuals’ self-reported uniqueness is an important factor to consider in persuasion. In Study 1, perceptions of thought uniqueness increased attitude-intention correspondence, an important indicator of an attitude’s durability. A second study demonstrated that recalling previous motivations to be unique also increased resistance to persuasion. Thus, even at the conceptual level, it seems that there is an inherent connection between uniqueness and resistance to influence (Tepper and Hoyle, 1996).

Nevertheless, the current studies were conducted under conditions of high personal relevance and motivation to counterargue. Future work should examine how perceptions of uniqueness are likely to resist under conditions of low involvement. Based on an Elaboration Likelihood Model perspective, less effortful resistance processes would likely occur under those conditions (Wegener et al., 2004). For example, perceptions of a thought’s uniqueness may serve as a cue that one has resisted the message, but the content of those thoughts may not actually be responsible for the resistance.^9^

It should be noted that, because only post-message attitudes were measured, it is unclear how much participants’ attitudes may have changed. Similarly, while we chose a topic that has been demonstrated as counterattitudinal in past studies (e.g., Cacioppo et al., 1984), some participants may have been initially favorable to the issue of comprehensive final exams. Future work may address these issues by including a pre-attack measure of attitudes and including only participants with initially unfavorable opinions.

One notable limitation of Study 2 is also that counterarguments were presented prior to the uniqueness manipulation. As mentioned earlier, this decision was consistent with Study 1’s design, such that uniqueness salience was introduced after the counterargumentation task. Unfortunately, doing so may have “decoupled” counterarguments from both attitudes and intentions, with the uniqueness manipulation being the primary driver of both attitudes and intentions. Indeed, the correlation between counterarguments and attitudes in Study 2 was *r* = 0.06 (*p* = 0.4), and counterarguments and intentions was *r* = 0.004 (*p* = 0.9). This also leads us to rule out self-validation as responsible for the effects in Study 2 (see Briñol and Petty, 2009), as the manipulation would serve to magnify the influence of counterarguments on attitudes and intentions. We would see this as a uniqueness x argument quality interaction in attitudes and intentions.

While the current work extends research on uniqueness and persuasion, future studies should explore the mechanisms for how uniqueness confers resistance. One possibility is that, from a metacognitive perspective, a sense of uniqueness may boost perceived veracity of one’s thoughts (Clarkson et al., 2013). Therefore, research examining the veracity-based properties of uniqueness may provide insight to a mechanism for the effects of current work and outline a limiting condition for these effects.

Increases in certainty may also only occur to the extent that uniqueness is viewed as positive. Uniqueness and autonomy are commonly considered positive aspects of the self in Western (individualistic) cultures, and the current studies included participants primarily from an individualistic culture. However, in more collectivistic cultures, a greater value is placed on fitting in or going along with the group (Markus and Kitayama, 1991). We believe that manipulations that highlight interdependent or collective aspects of the self may lead to increased resistance to persuasion in collectivistic cultures. In collectivistic cultures where the interdependent self is valued, learning that one’s counterarguments are common or typical may actually lead to greater resistance than in individualistic cultures, whereas generating unique thoughts when similarity is made salient may decrease persuasion. We look forward to examining such a possibility in the future.

As the earliest (Yale School) empirical studies of attitude change demonstrated, persuasion is never one-size-fits-all. Research in persuasion must always consider characteristics of message receivers and the circumstances under which messages are delivered when determining the antecedents of strong attitudes and willingness to act upon those attitudes. These studies therefore have implications for understanding why a need for uniqueness leads some individuals to literally risk their lives by refusing to wear seatbelts or masks, while inspiring others to seek positive uniqueness through altruistic actions such as volunteering to help family members during a health crisis. As our understanding of uniqueness and attitude intentions grows, so too will our ability to persuade others to engage in community-oriented courses of action.

## Data Availability Statement

The raw data supporting the conclusions of this article will be made available by the authors, without undue reservation.

## Ethics Statement

The studies involving human participants were reviewed and approved by Institutional Review Board of Iowa State University. The patients/participants provided their written informed consent to participate in this study.

## Author Contributions

KB and KK developed the idea, designed the studies, and wrote the manuscript with feedback and edits from MM. KB conducted and analyzed the studies with feedback from KK and MM. All authors contributed to the article and approved the submitted version.

## Conflict of Interest

The authors declare that the research was conducted in the absence of any commercial or financial relationships that could be construed as a potential conflict of interest.

## Footnotes

1 We conducted a sensitivity analysis and determined that a small effect size (Cohen’s *d* = 0.18) could be detected with *N* = 88 at 80% power.

2 Participants completed a series of filler questionnaires, including a 35-item behavior inventory, the Need to Evaluate scale (Jarvis and Petty, 1996), the Need for Cognition scale (Cacioppo et al., 1984), and the Need to Belong scale (Leary et al., 2013) just prior to completing the demographic measure.

3 We use the term counterarguments to mean unfavorable thoughts toward the message, which is consistent with previous research (Petty and Cacioppo, 1977; Cacioppo et al., 1981).

4 One could also include participants’ counterarguments in a moderated mediation analysis, with attitudes mediating the influence of counterarguments on willingness, and that mediation being moderated by perceptions of thought uniqueness. To test this, we conducted the analysis using the PROCESS macro for SPSS (Model 14; Hayes, 2013). Results revealed no moderation of the indirect effects from thoughts to willingness was significant (all 95% CIs included 0). However, the attitude x counterargument uniqueness interaction was significant *b* = −0.19, *SE* = 0.08, *t*(83) = −2.63, *p* = 0.01, 95% CI [−0.35, −0.05] and in the same pattern as reported in the text. Perhaps the lack of moderated mediation is twofold: lack of power for such a complex analysis, and introduction of the uniqueness salience after thoughts were generated.

5 Including recall enjoyment as a covariate in the ANOVA analyses did not change the significance of any of the reported effects.

6 Decomposition of the interaction at each level of the uniqueness variable revealed that the difference in argument quality on counterarguments was larger in the uniqueness conditions that the fit-in conditions. Thus, counterarguments did show a pattern consistent with previous literature, despite the interaction on attitudes being nonsignificant.

7 A follow-up analysis revealed the purchase x attitude x argument quality interaction was marginally significant *b* = 0.08 *SE* = 0.05, *t*(226) = 10.72, *p* = 0.09, 95% *CI* [−0.01, 0.17]. Decomposition of the interaction at each level of argument quality revealed that the purchase x attitude interaction was significant in the weak argument conditions *b* = −0.14, *SE* = 0.06, *t*(115) = −2.33, *p* = 0.02, 95% *CI* [−0.26, −0.02], but not the strong *b* = 0.01, *SE* = 0.07, *t*(111) = 0.2, *p* = 0.84 argument conditions. Thus, consistent with Study 1, attitudes were more strongly related to willingness at high levels of counterargument uniqueness in the weak argument conditions.

8 Because we found an unexpected effect of argument quality moderating the purchase x attitude interaction on intentions (see footnote 6), we also conducted a moderated mediation analysis to examine whether argument quality moderated the mediation of attitudes on the uniqueness to willingness link. We thus conducted a moderated mediation using the PROCESS macro (Model 7). Results revealed that while the indirect effect of attitudes mediating the purchase manipulation on willingness was stronger in the weak argument conditions (indirect effect *M* = 0.2, *SE* = 0.08) than in the strong argument conditions (indirect effect *M* = 0.09, *SE* = 0.09), the index of moderated mediation was not significant (i.e., the bootstrapped 95% CI contained 0).

9 Readers may be wondering why there was no effect of argument quality on counterarguments in Study 2, as there had been with attitudes and intentions. One possibility may be because of guided nature of the instructions provided to participants. In much of the persuasion research participants are asked to report their thoughts about the message (Petty and Cacioppo, 1986), which likely does not constrain the favorability of the thoughts. In the current studies, participants were explicitly asked to create counterarguments, which may have biased the thoughts to be negative and likely restricting the range of thought favorability and creating a disconnect between thoughts and attitudes. Moreover, unlike thoughts, participants were not asked to report negative attitudes. This may be partly responsible for the lack of argument quality effect on thoughts.
